# Transradial Renal Angioplasty and Stent Placement for Systemic Hypertension Caused by Severe Unilateral Renovascular Stenosis

**DOI:** 10.7759/cureus.34781

**Published:** 2023-02-08

**Authors:** Christopher A Hebert, Robert L Rosenthal, Jeffrey M Schussler

**Affiliations:** 1 Nephrology, Baylor University Medical Center, Dallas, USA; 2 Cardiology, Baylor University Medical Center, Dallas, USA; 3 Cardiology, Baylor Scott & White Heart and Vascular Hospital, Dallas, USA; 4 Interventional Cardiology, Baylor Scott & White Heart and Vascular Hospital, Dallas, USA

**Keywords:** percutaneous transluminal peripheral angioplasty, peripheral vascular disease, renal artery stenting, atherosclerotic renal artery, transradial access, renovascular hypertension

## Abstract

Percutaneous transluminal angioplasty and stent placement for renovascular hypertension is a recognized albeit seldom used therapy. We present a case of severe renovascular hypertension, due to renal artery atherosclerosis, treated successfully with stent placement via the radial artery access approach.

## Introduction

We present a case of severe secondary hypertension in a young woman due to renal artery atherosclerosis, successfully treated through percutaneous transluminal angioplasty. This case highlights several issues pertinent to physicians; Renal artery atherosclerosis as a secondary cause of malignant hypertension, the use of magnetic resonance as an imaging modality to evaluate for renal artery stenosis, and the use of the transradial approach for successful renal artery intervention.

## Case presentation

A 44-year-old woman with historically normal blood pressure (100/60 average), acceptable cholesterol (LDL 111 mg/dl), no family history of early cardiovascular disease, BMI 27 and no personal prior cardiovascular history began to have headaches associated with elevated blood pressures in the 180 systolic range. She was started on a beta blocker and ace-inhibitor and referred to a cardiologist for severe “essential hypertension.”

Over a three-month period of time, her blood pressure became increasingly difficult to control despite attempts to control it with multiple medications (beta blockers, ace-inhibitors, thiazide diuretics, and ultimately aldosterone antagonists).

**Figure 1:**
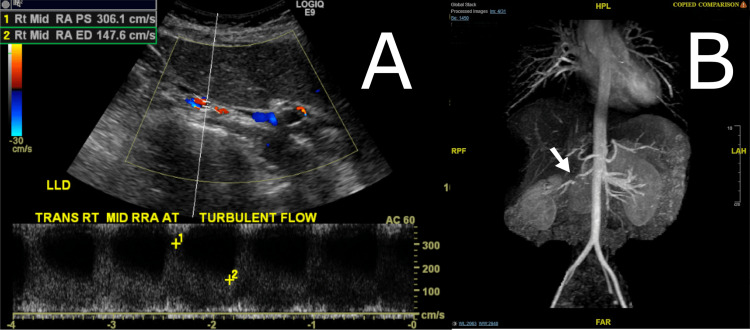
Renal artery Doppler and magnetic resonance angiogram Doppler interrogation of the right renal artery (Panel A) showing increased velocities and turbulent flow, suggestive of a hemodynamically significant stenosis. (Panel B) Magnetic resonance angiography of the aorta and renal vasculature, demonstrating a severe, focal stenosis of the right renal artery.

Given her young age and the accelerated course of her hypertension, secondary causes were considered. Renal artery Doppler and ultrasound (Figure 1A) suggested severe right renal artery stenosis: the right kidney was smaller (9.4 cm in length) than the left kidney (11.7 cm) with an elevated peak systolic velocity of 306 cm/s and turbulent flow distally in the artery.

Magnetic resonance imaging (Figure 1B) demonstrated a focal, critical stenosis in the right renal artery, and she was then set up for invasive angiography.

**Figure 2:**
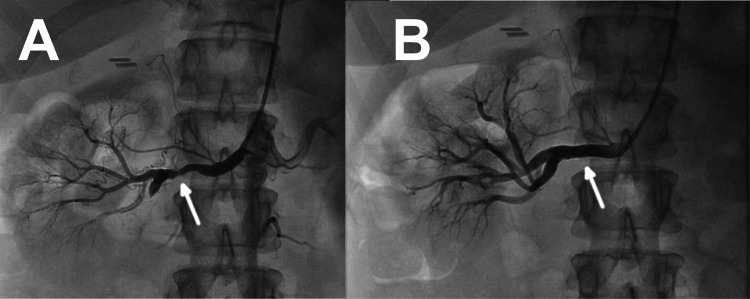
Renal artery angiogram: pre- and post-angioplasty and stent placement Right renal angiogram performed using multipurpose guide demonstrating a high grade, atherosclerotic plaque in the mid portion of the artery (panel A). Post stent placement, there is normal flow into the inferior branch off of the renal artery, and no residual stenosis (panel B).

Diagnostic renal angiography was performed using a 5Fr Judkins right catheter through a right radial access approach. It confirmed a severe stenosis of the right renal artery (Figure 2A) and a normal left renal artery. The angiographic appearance of the stenosis was consistent with atherosclerosis, with obvious reduction of flow into portions of the distal vasculature.

Percutaneous transluminal angioplasty and stent placement was performed using a 100 cm multipurpose guide. The lesion was ballooned with a 2.5 x 12 mm balloon, and then stented with a 5.0 mm x 16 mm Synergy Megatron (Boston Scientific, Marlborough, MA) drug eluting stent, deployed at 16 atmospheres (Figure 2B). The type of stent was chosen due to sizing and availability, and not specifically due to any (theoretical) considerations of its drug-eluting properties.

The patient’s blood pressure, which was 179/113 on an ER visit the week prior, was 121/80 3 hours post procedure prior to discharge. Within two weeks she was off all anti-hypertensive medications.

She remains, over one year later, normotensive off of all medications.

## Discussion

Renovascular hypertension makes up about 5% of cases of all hypertension cases, atherosclerotic renal arterial disease and fibromuscular dysplasia representing the most significant groups. In both of those groups, there has been evidence that percutaneous transluminal renal angioplasty and stenting can improve blood pressure, reduce flash pulmonary edema, and prevent worsening of renal dysfunction [1]. Currently, there is a dearth of randomized controlled trials to support this, but it is recognized as an appropriate therapy for unilateral (or bilateral) renal artery stenosis in the setting of accelerated/resistant hypertension [2].

Transradial access for renal artery interventions is a recognized, although less-often used approach. As with coronary interventions, it has the potential to reduce access site complications, reduce post-procedure bedrest, and improve patient satisfaction, while still allowing successful intervention [3].

Due to the angulation of the renal arteries, a cranio-caudal approach may be advantageous in that approaching from above the renal arteries may improve cannulation and support of the delivery of equipment [4]. Some potential drawbacks or considerations need to be observed, in that radial access may require the use of equipment with longer lengths, and that in taller patients there may be difficulty reaching the renal arteries. In some patients, removal of intervening equipment (backflow valves) or utilizing the left-radial approach may be necessary [5].

## Conclusions

We present this case as an example of secondary renovascular hypertension, successfully treated with mechanical correction of a severe atherosclerotic stenosis of the renal artery. With renal artery denervation on the horizon as a therapy for hypertension, transradial access for percutaneous renal artery therapies may be an area of increased interest. This case also highlights the fact that even in patients without significant risk factors, pursuing a diagnosis based on clinical findings is important and can result in significant and beneficial treatment outcomes.
